# Survey of Hfe Gene C282Y Mutation in Turkish Beta-Thalassemia Patients and Healthy Population: A Preliminary Study

**DOI:** 10.4274/tjh.2012.0081

**Published:** 2014-09-05

**Authors:** Selma Ünal, Günay Balta, Fatma Gümrük

**Affiliations:** 1 Mersin University Faculty of Medicine, Department of Pediatric Hematology, Mersin, Turkey; 2 Hacettepe University Faculty of Medicine, Department of Pediatric Hematology, Ankara, Turkey

**Keywords:** Beta-thalassemia, C282Y mutation, Hemochromatosis

## Abstract

**Objective:** This study was planned in order to determine the effect of C282Y mutation in development of secondary hemochromatosis in beta-thalassemia patients and to determine the prevalence and allele frequency of this mutation in a healthy control group.

**Materials and Methods:** Eighty-seven children and young adults (46 males and 41 females; mean age: 15.6±6.1 years, range: 3-30 years) with beta-thalassemia major (BTM) and 13 beta-thalassemia intermedia (BTI) patients (6 males and 7 females; mean age: 19.6±3.5 years, range: 13-26 years) were included in the study. The control group comprised 100 healthy blood donors.

**Results:** Neither heterozygous nor homozygous HFE gene C282Y mutation was detected in patients with BTM or BTI, or in control group.

Conclusion: The C282Y mutation, which is supposed to be responsible for the majority of hereditary hemochromatosis, was not found to have a role in the development of hemochromatosis in beta-thalassemia patients and was not detected in a healthy Turkish population. However, research on larger cohorts of individuals is required in order to determine the exact prevalence of the HFE gene mutation in Turkish populations from diverse ethnic origins and whether it would have an impact on iron loading in thalassemic populations.

## OZET

**Amaç:** Herediter hemokromatozisin büyük bir kısmından sorumlu tutulan C282Y mutasyonunun beta talasemili hastalarda gelişen sekonder hemokromatozisdeki arttırıcı rolünü belirlemek ve bu mutasyonun, sağlıklı kontrol grubunda prevalansını ve allel sıklığını göstermek.

**Gereç ve Yöntemler:** Kırk biri kız, 46’si erkek (ortanca yaş: 15,6±6,1, yaş aralığı: 3-30 yaş) toplam 87 Beta talasemi major ve 13 Beta talasemi intermedialı hasta (6 erkek, 7 kız, ortanca yaş: 19,6±3,5, yaş aralığı: 13-26) çalışmanın hasta grubunu oluşturdu. Kontrol grubu olarak 100 sağlıklı birey çalışmaya dahil edildi.

Bulgular: Ne Beta talasemili hasta ne de sağlıklı bireyde homozigot yada heterezigot C282Y mutasyonu tespit edilemedi.

**Sonuç:** Herediter hemokromatozis gelişiminde önemli bir faktör olarak düşünülen C282Y mutasyonu, sağlıklı Türk popülasyonu ve Beta talasemili hastalarda hemokromatozis gelişimi için arttırıcı bir faktör olarak bulunamamıştır. Bu konuda daha geniş katılımlı çalışmaların yapılmasına gerek olup, demir metabolizmasından sorumlu olan diğer düzenleyici protein gen mutasyonlarının da sekonder hemokromatozisi olan beta talasemili hastalarda araştırılması gerektiği düşünülmektedir.

## INTRODUCTION

Hereditary hemochromatosis (HH) is a disease of iron regulation that results in excessive iron absorption and ultimately in iron overload and multiple organ failure, such as cirrhosis of the liver, diabetes mellitus, arthropathy, and cardiomyopathy [1,2]. In northern Europe, HH affects approximately 1 in 200-300 persons [3]. This autosomal recessive condition stems from at least 2 genetic mutations in the HFE gene (also known as the HLA-H gene), a candidate gene localized to chromosome 6p22.1. A G-to-A transition at nucleotide 845 (845 A-G) results in a Cys-to-Tyr substitution at codon 282 (C282Y), whereas a C-to-G change at position 187 causes a His-to-Asp mutation at codon 63 (H63D) [4,5]. There is a clear association between C282Y and HH. Over 90% of HH patients from the UK are homozygous for this mutation [6]. However, in Italy and France, 70% of HH cases are homozygous for C282Y [7,8].

Beta-thalassemia is a serious genetic disorder that increases iron absorption, and regular blood transfusion causes iron accumulation and secondary hemochromatosis. Excess iron is extremely toxic to all cells of the body and can cause serious and irreversible organic damage, such as cirrhosis, diabetes, heart disease, and hypogonadism [9]. While the causes of iron accumulation in beta-thalassemia patients are clear, the fact that this accumulation occurs in higher amounts in some patients suggests a possible association between HH and beta-thalassemia. This study was planned in order to determine the effect of the C282Y mutation, which is supposed to be responsible for the majority of HH, in the development of secondary hemochromatosis in beta-thalassemia patients, and to determine the prevalence and allele frequency of this mutation in a healthy control group.

## MATERIALS AND METHODS

Eighty-seven children and young adults (46 males and 41 females; mean age: 15.6±6.1 years, range: 3-30 years) with beta-thalassemia major (BTM) and 13 beta-thalassemia intermedia (BTI) patients (6 males and 7 females; mean age: 19.6±3.5 years, range: 13–26 years) who were followed in the Pediatric Hematology Unit of Hacettepe University, Ankara, Turkey, were included in the study. One hundred healthy blood donors who were consecutively selected from the same geographic area of Turkey without personal or family history of hemochromatosis constituted the control group. This study was approved by the Hacettepe University Ethics Board and informed consent was obtained from all subjects included in the study.

A full physical examination was performed and a detailed medical history was taken for every patient. The diagnosis of thalassemia was made based on the clinical presentation, hematological indices including hemoglobin electrophoresis, and mutation analysis. Hemochromatosis was evaluated based on serum ferritin, alanine aminotransferase (ALT), and aspartate aminotransferase (AST) levels in all the beta-thalassemia patients by standard methods. 

**Analysis of HFE Gene C282Y Mutation**

Genomic DNA was isolated from 10 mL of peripheral blood by the standard manual methods of phenol-chloroform extraction. PCR amplification of the DNA region encompassing the C282Y mutation site was performed by using the primer pair reported before (F5’CAAGTGCCTCCTTTGGTGAAGGTGACACAT3’; R5’CTCAGGCACTCCTCTCAACC3’) at the stepwise annealing temperatures of 60 °C (5 cycles) and 64 °C (30 cycles) [3]. Rsa I digestion of 343-bp PCR products was expected to yield 203- and 140-bp DNA fragments for normal alleles and 203-, 111-, and 29-bp DNA fragments for mutant alleles.

## RESULTS

The mean ALT, AST, and ferritin levels were 46.3 IU (range: 9-150), 43 IU (range: 17-154), and 3166 µg/L (range: 890-16,000) in the patients with BTM and 38 IU (range: 11-85), 41 IU (range: 14-87), and 718 µg/L (range: 163-1689) in the patients with BTI, respectively. According to the beta-globin gene mutation analysis, IVS-I-110/IVS-I-110 was the most common mutation in BTM patients (27%). Neither heterozygous nor homozygous HFE gene C282Y mutation was detected in patients with BTM and BTI or in the control group.

## DISCUSSION

In Mediterranean countries, the most common cause of iron overload is BTM. Iron accumulation in thalassemic patients depends both on increased intestinal iron absorption, which is proportional to the degree of erythroid hyperplasia, and on blood transfusions. Progress in iron-chelating therapy over the last 20 years has dramatically changed the prognosis of these patients, as iron overload may be maintained at low levels in regularly transfused thalassemic subjects by applying lifelong regular chelation [10,11]. While the causes of iron accumulation in beta-thalassemia patients are obvious, the fact that this accumulation occurs in higher amounts in some patients suggests a possible relation between the gene mutations that cause beta-thalassemia and HH. HH is an autosomal recessive disease characterized by abnormal accumulation of iron in parenchymal organs and organ failures [1]. It has been shown that 2 mutations, which were identified after isolation of the HFE gene, are responsible for the majority of the cases. Among these mutations, the homozygous C282Y mutation is present in the etiology of a major proportion of HH patients [4]. The effect of the HFE gene C282Y mutation of hemochromatosis in the occurrence of non-transfusional iron overload in patients with beta-thalassemia has been controversial.

In previous reports from different countries, the association between beta-thalassemia and the HFE gene mutation has been investigated. In India, thalassemia is the most common cause of iron overload while HH is very rare. Severe iron overload was reported in a 61-year-old Indian patient with BTI, heterozygous for C282Y [12]. This finding allowed speculation that defective HFE might behave as a modifying gene involved in the severity of iron loading in beta-thalassemia. Piperno et al. suggested that the beta-thalassemia trait aggravates the clinical picture of C282Y homozygotes, favoring higher rates of iron accumulation and the development of severe iron-related complications [13]. In a study by Jazayeri et al., heterozygote C282Y mutation was found in 3.2% of patients with the beta-thalassemia trait. However, the mean ferritin level in patients with HFE mutations showed no significant difference from that of patients without mutations [14].

Longo et al. detected that the allele frequency of C282Y mutation was 1.4% in Italian thalassemics and they suggested that the presence of a single mutation in the HFE gene did not influence the severity of iron loading in thalassemia patients [15].

We did not detect the C282Y mutation in the Turkish beta-thalassemia patients or the controls. Similarly, the C282Y mutation was not detected in healthy blood donors in 2 previous studies from Turkey [16,17]. In contrast, a homozygous C282Y mutation was detected by Yönal et al. in a Turkish family [18].

In conclusion, this study did not support the role of C282Y mutation in the development of secondary hemochromatosis in a group of beta-thalassemia patients. 

However, further research on larger cohorts of individuals is required in order to determine the exact prevalence of HFE gene mutation in Turkish populations from diverse ethnic origins and whether or not this would have an impact on iron loading in thalassemic populations.

The role of genetic factors other than the C282Y mutation contributing to hemochromatosis should also be studied in the thalassemic population with severe iron overload despite good compliance to chelating regimens in Turkey.

**Acknowledgment**

The authors would like to thank Prof. Dr. Aytemiz Gürgey for her invaluable comments and advice.

**Conflict of Interest Statement**

The authors of this paper have no conflicts of interest, including specific financial interests, relationships, and/ or affiliations relevant to the subject matter or materials included.
